# 9,9-Dimethyl-9-silafluorene

**DOI:** 10.1107/S1600536809003614

**Published:** 2009-02-04

**Authors:** Jan Mewes, Hans-Wolfram Lerner, Michael Bolte

**Affiliations:** a Institut für Anorganische Chemie, J. W. Goethe-Universität Frankfurt, Max-von-Laue-Strasse 7, 60438 Frankfurt/Main, Germany

## Abstract

The title compound, C_14_H_14_Si, crystallizes with two almost identical mol­ecules (r.m.s. deviation = 0.080 Å for all non-H atoms) in the asymmetric unit. All atoms of the silafluorene moiety lie in a common plane (r.m.s. deviations = 0.049 and 0.035 Å for the two mol­ecules in the asymmetric unit). The Si—C_meth­yl_ bonds are significantly shorter [1.865 (4)–1.868 (4) Å] than the Si—C_aromatic_ bonds [1.882 (3)–1.892 (3) Å]. Owing to strain in the five-membered ring, the endocyclic C—Si—C angles are reduced to 91.05 (14) and 91.21 (14)°.

## Related literature

For the synthesis, see: Hudrlik *et al.* (2006 ▶). For related compounds, see: Kaufmann *et al.* (2008 ▶).
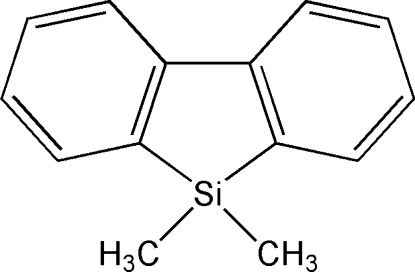


## Experimental

### 

#### Crystal data

C_14_H_14_Si
*M* *_r_* = 210.34
Monoclinic, 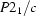

*a* = 16.1336 (8) Å
*b* = 8.7752 (5) Å
*c* = 17.0227 (11) Å
β = 92.208 (5)°
*V* = 2408.2 (2) Å^3^
*Z* = 8
Mo *K*α radiation
μ = 0.16 mm^−1^
*T* = 173 (2) K
0.22 × 0.17 × 0.09 mm

#### Data collection

Stoe IPDS-II two-circle diffractometer
Absorption correction: multi-scan (*MULABS*; Spek, 2003 ▶; Blessing, 1995 ▶) *T*_min_ = 0.966, *T*_max_ = 0.976
38382 measured reflections
4404 independent reflections
3274 reflections with *I* > 2σ(*I*)
*R*_int_ = 0.082

#### Refinement

*R*[*F* ^2^ > 2σ(*F* ^2^)] = 0.061
*wR*(*F* ^2^) = 0.157
*S* = 1.12
4404 reflections
271 parameters
H-atom parameters constrained
Δρ_max_ = 0.38 e Å^−3^
Δρ_min_ = −0.29 e Å^−3^

### 

Data collection: *X-AREA* (Stoe & Cie, 2001 ▶); cell refinement: *X-AREA*; data reduction: *X-AREA*; program(s) used to solve structure: *SHELXS97* (Sheldrick, 2008 ▶); program(s) used to refine structure: *SHELXL97* (Sheldrick, 2008 ▶); molecular graphics: *XP* in *SHELXTL-Plus* (Sheldrick, 2008 ▶); software used to prepare material for publication: *SHELXL97* and *PLATON* (Spek, 2003 ▶).

## Supplementary Material

Crystal structure: contains datablocks I, global. DOI: 10.1107/S1600536809003614/at2718sup1.cif

Structure factors: contains datablocks I. DOI: 10.1107/S1600536809003614/at2718Isup2.hkl

Additional supplementary materials:  crystallographic information; 3D view; checkCIF report

## Figures and Tables

**Fig. 1.**
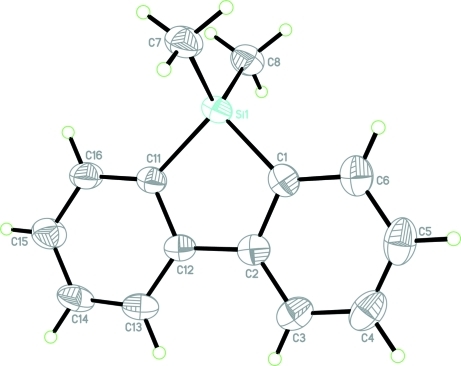
Perspective view of the first molecule in the asymmetric unit of the title compound with the atom numbering scheme; displacement ellipsoids are at the 50% probability level; H atoms are drawn as small spheres of arbitrary radii.

**Fig. 2.**
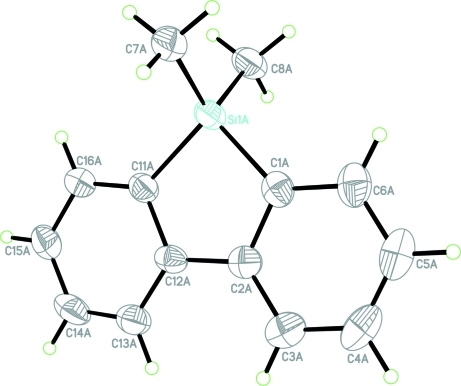
Perspective view of the second molecule in the asymmetric unit of the title compound with the atom numbering scheme; displacement ellipsoids are at the 50% probability level; H atoms are drawn as small spheres of arbitrary radii.

**Fig. 3.**
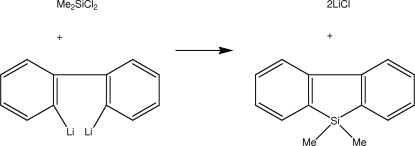
The formation of the title compound.

## supplementary crystallographic information

### Comment

Our group has a long-standing interest in redox-switchable Lewis acids derived from ferrocenylboranes. Very recently we have synthesized 9-ferrocenyl-9-borafluorene derivatives (Kaufmann *et al.*, 2008). Herein we describe the preparation and structure of 9,9-dimethyl-9-silafluorene (Me_2_SiC_12_H_8_) which was used as a starting material in the synthesis of these 9-ferrocenyl-9-borafluorene derivatives. The title compound was obtained by treatment of 2,2'-dilithio biphenylene with dichlorodimethylsilane Me_2_SiCl_2_ following a literature procedure (Hudrlik *et al.*, 2006) as indicated in the equation below.

The title compound crystallizes with two almost identical molecules (r.m.s. deviation 0.080Å for all non-H atoms except the methyl groups) in the asymmetric unit (Fig. 1 and 2). All atoms of the silafluorene moiety lie in a common plane (r.m.s. deviation 0.049 Å and 0.035 Å for the two molecules in the asymmetric unit. The Si—C_methyl_ bonds are considerably shorter [1.865 (4) Å to 1.868 (4) Å] than the Si—C_aromatic_ bonds [1.882 (3) Å to 1.892 (3) Å]. Due to the strain in the five-membered ring, the innercyclic C—Si—C angle is reduced to 91.05 (14)° and 91.21 (14)°, respectively.

### Experimental

A solution of Me_2_SiCl_2_ (1.9 ml, 2.03 g, 15.8 mmol) and NEt_3_ (1.9 ml, 1.38 g, 13.6 mmol) in 50 ml THF was added dropwise to a solution of 2,2'-dilithio biphenylene (14.6 mmol) which was generated from one equivalent of 2,2'-dibrom biphenylene (4.46 g, 14.6 mmol) and two equivalents of *n*BuLi (28.8 mmol) in 50 ml diethyl ether at 195 K. After stirring for 2 h the solution was filtered. After removing the solvent, the residue was treated with 50 ml water and 30 ml diethyl ether. After removing the diethyl ether from the organic layer, X-ray quality crystals were obtained by sublimation (80%). The NMR spectra were recorded on a Bruker DPX 250 and a Bruker avance 400 spectrometer. 9,9-dimethyl-9-silafluorene: ^1^H NMR (CDCl_3_, internal TMS): δ 0.43 (s; 6 H, MeSi), δ 7,28 (m; 2 H), δ 7.44 (m; 2 H), δ 7.64 (m; 2 H), δ 7.83 (m; 2 H). ^13^C{^1^H}NMR (CDCl_3_, internal TMS): δ -3.2 (MeSi), δ 120.8, δ 127.4, δ 130.2, δ 132.7, δ 139.0, δ 140.9.

### Refinement

H atoms were geometrically positioned and refined using a riding model with fixed individual displacement parameters [U_iso_(H) = 1.2 *U*_eq_(C) or U_iso_(H) = 1.5 *U*_eq_(C_methyl_)] using a riding model with C—H(aromatic) = 0.95 Å or C—H(methyl) = 0.98 Å, respectively.

### Crystal data

**Table:**

C_14_H_14_Si	*F*(000) = 896
*M**_r_* = 210.34	*D*_x_ = 1.160 Mg m^−^^3^
Monoclinic, *P*2_1_/*c*	Mo *K*α radiation, λ = 0.71073 Å
Hall symbol: -P 2ybc	Cell parameters from 18030 reflections
*a* = 16.1336 (8) Å	θ = 2.4–27.5°
*b* = 8.7752 (5) Å	µ = 0.16 mm^−^^1^
*c* = 17.0227 (11) Å	*T* = 173 K
β = 92.208 (5)°	Plate, colourless
*V* = 2408.2 (2) Å^3^	0.22 × 0.17 × 0.09 mm
*Z* = 8	

### Data collection

**Table:**

Stoe IPDS-II two-circle diffractometer	4404 independent reflections
Radiation source: fine-focus sealed tube	3274 reflections with *I* > 2σ(*I*)
graphite	*R*_int_ = 0.082
ω scans	θ_max_ = 25.4°, θ_min_ = 2.4°
Absorption correction: multi-scan (*MULABS*; Spek, 2003; Blessing, 1995)	*h* = −19→19
*T*_min_ = 0.966, *T*_max_ = 0.976	*k* = −10→10
38382 measured reflections	*l* = −20→20

### Refinement

**Table:**

Refinement on *F*^2^	Primary atom site location: structure-invariant direct methods
Least-squares matrix: full	Secondary atom site location: difference Fourier map
*R*[*F*^2^ > 2σ(*F*^2^)] = 0.061	Hydrogen site location: inferred from neighbouring sites
*wR*(*F*^2^) = 0.157	H-atom parameters constrained
*S* = 1.12	*w* = 1/[σ^2^(*F*_o_^2^) + (0.0668*P*)^2^ + 1.3736*P*] where *P* = (*F*_o_^2^ + 2*F*_c_^2^)/3
4404 reflections	(Δ/σ)_max_ < 0.001
271 parameters	Δρ_max_ = 0.38 e Å^−^^3^
0 restraints	Δρ_min_ = −0.29 e Å^−^^3^

### Special details

**Table:**

Geometry. All e.s.d.'s (except the e.s.d. in the dihedral angle between two l.s. planes) are estimated using the full covariance matrix. The cell e.s.d.'s are taken into account individually in the estimation of e.s.d.'s in distances, angles and torsion angles; correlations between e.s.d.'s in cell parameters are only used when they are defined by crystal symmetry. An approximate (isotropic) treatment of cell e.s.d.'s is used for estimating e.s.d.'s involving l.s. planes.
Refinement. Refinement of *F*^2^ against ALL reflections. The weighted *R*-factor *wR* and goodness of fit *S* are based on *F*^2^, conventional *R*-factors *R* are based on *F*, with *F* set to zero for negative *F*^2^. The threshold expression of *F*^2^ > σ(*F*^2^) is used only for calculating *R*-factors(gt) *etc*. and is not relevant to the choice of reflections for refinement. *R*-factors based on *F*^2^ are statistically about twice as large as those based on *F*, and *R*- factors based on ALL data will be even larger.

### Fractional atomic coordinates and isotropic or equivalent isotropic displacement parameters (Å^2^)

**Table:**

	*x*	*y*	*z*	*U*_iso_*/*U*_eq_	
Si1A	0.04143 (6)	0.84574 (10)	0.17553 (5)	0.0343 (2)	
C1A	0.09531 (19)	0.8679 (4)	0.27489 (19)	0.0346 (7)	
C2A	0.15535 (19)	0.9860 (4)	0.27160 (19)	0.0336 (7)	
C3A	0.2043 (2)	1.0224 (4)	0.3386 (2)	0.0428 (8)	
H3A	0.2449	1.1007	0.3365	0.051*	
C4A	0.1936 (3)	0.9439 (5)	0.4081 (2)	0.0515 (10)	
H4A	0.2274	0.9681	0.4534	0.062*	
C5A	0.1342 (3)	0.8308 (5)	0.4124 (2)	0.0515 (10)	
H5A	0.1272	0.7782	0.4605	0.062*	
C6A	0.0847 (2)	0.7941 (4)	0.3465 (2)	0.0439 (8)	
H6A	0.0433	0.7178	0.3501	0.053*	
C7A	0.0576 (2)	0.6583 (4)	0.1269 (2)	0.0466 (9)	
H7A1	0.1172	0.6368	0.1256	0.070*	
H7A2	0.0339	0.6616	0.0730	0.070*	
H7A3	0.0303	0.5780	0.1563	0.070*	
C8A	−0.0723 (2)	0.8848 (4)	0.1776 (2)	0.0458 (9)	
H8A1	−0.0813	0.9832	0.2032	0.069*	
H8A2	−0.0993	0.8040	0.2070	0.069*	
H8A3	−0.0958	0.8878	0.1237	0.069*	
C11A	0.10420 (19)	1.0065 (4)	0.13428 (19)	0.0326 (7)	
C12A	0.15905 (18)	1.0648 (4)	0.19395 (19)	0.0321 (7)	
C13A	0.2105 (2)	1.1889 (4)	0.1784 (2)	0.0408 (8)	
H13A	0.2475	1.2273	0.2184	0.049*	
C14A	0.2074 (2)	1.2558 (4)	0.1047 (2)	0.0467 (9)	
H14A	0.2415	1.3412	0.0947	0.056*	
C15A	0.1546 (2)	1.1985 (4)	0.0452 (2)	0.0441 (9)	
H15A	0.1532	1.2442	−0.0054	0.053*	
C16A	0.1037 (2)	1.0741 (4)	0.0600 (2)	0.0387 (8)	
H16A	0.0683	1.0348	0.0190	0.046*	
Si1	0.46279 (5)	0.38341 (10)	0.17151 (5)	0.0319 (2)	
C1	0.40267 (19)	0.4048 (4)	0.26355 (19)	0.0328 (7)	
C2	0.34203 (18)	0.5214 (4)	0.25218 (19)	0.0319 (7)	
C3	0.2873 (2)	0.5548 (4)	0.3116 (2)	0.0396 (8)	
H3	0.2464	0.6318	0.3037	0.048*	
C4	0.2928 (2)	0.4752 (5)	0.3820 (2)	0.0458 (9)	
H4	0.2551	0.4971	0.4219	0.055*	
C5	0.3532 (3)	0.3635 (4)	0.3947 (2)	0.0488 (9)	
H5	0.3569	0.3104	0.4433	0.059*	
C6	0.4083 (2)	0.3298 (4)	0.3358 (2)	0.0418 (8)	
H6	0.4501	0.2549	0.3451	0.050*	
C7	0.5764 (2)	0.4193 (4)	0.1862 (2)	0.0420 (8)	
H7A	0.5852	0.5171	0.2130	0.063*	
H7B	0.6022	0.4222	0.1351	0.063*	
H7C	0.6015	0.3375	0.2183	0.063*	
C8	0.4471 (2)	0.1964 (4)	0.1206 (2)	0.0432 (8)	
H8A	0.3876	0.1767	0.1126	0.065*	
H8B	0.4724	0.1151	0.1529	0.065*	
H8C	0.4732	0.1994	0.0695	0.065*	
C11	0.40308 (18)	0.5457 (4)	0.12296 (18)	0.0312 (7)	
C12	0.34395 (18)	0.6015 (4)	0.17523 (18)	0.0297 (7)	
C13	0.2947 (2)	0.7273 (4)	0.1539 (2)	0.0383 (8)	
H13	0.2552	0.7648	0.1890	0.046*	
C14	0.3034 (2)	0.7970 (4)	0.0819 (2)	0.0431 (8)	
H14	0.2698	0.8824	0.0679	0.052*	
C15	0.3610 (2)	0.7436 (4)	0.0297 (2)	0.0463 (9)	
H15	0.3670	0.7922	−0.0196	0.056*	
C16	0.4098 (2)	0.6178 (4)	0.0506 (2)	0.0414 (8)	
H16	0.4485	0.5803	0.0147	0.050*	

### Atomic displacement parameters (Å^2^)

**Table:**

	*U*^11^	*U*^22^	*U*^33^	*U*^12^	*U*^13^	*U*^23^
Si1A	0.0323 (5)	0.0327 (5)	0.0383 (5)	−0.0072 (4)	0.0058 (3)	−0.0003 (4)
C1A	0.0344 (16)	0.0312 (17)	0.0390 (18)	0.0049 (14)	0.0098 (13)	−0.0021 (14)
C2A	0.0286 (16)	0.0329 (17)	0.0395 (18)	0.0051 (13)	0.0035 (13)	−0.0057 (14)
C3A	0.0374 (18)	0.044 (2)	0.046 (2)	0.0087 (15)	−0.0037 (15)	−0.0084 (16)
C4A	0.064 (2)	0.055 (2)	0.035 (2)	0.018 (2)	−0.0069 (17)	−0.0116 (17)
C5A	0.068 (3)	0.053 (2)	0.0334 (19)	0.018 (2)	0.0061 (17)	0.0028 (16)
C6A	0.052 (2)	0.041 (2)	0.0398 (19)	0.0095 (16)	0.0133 (16)	0.0001 (15)
C7A	0.055 (2)	0.041 (2)	0.045 (2)	−0.0138 (17)	0.0127 (16)	−0.0058 (16)
C8A	0.0376 (18)	0.047 (2)	0.053 (2)	−0.0107 (16)	0.0045 (15)	0.0038 (17)
C11A	0.0283 (16)	0.0328 (17)	0.0370 (17)	−0.0029 (13)	0.0057 (13)	−0.0016 (13)
C12A	0.0223 (14)	0.0317 (17)	0.0424 (18)	0.0010 (12)	0.0036 (12)	−0.0069 (14)
C13A	0.0348 (18)	0.039 (2)	0.049 (2)	−0.0062 (14)	0.0047 (15)	−0.0071 (16)
C14A	0.0388 (19)	0.039 (2)	0.063 (2)	−0.0138 (16)	0.0102 (16)	0.0006 (18)
C15A	0.045 (2)	0.045 (2)	0.042 (2)	−0.0076 (16)	0.0103 (15)	0.0062 (16)
C16A	0.0334 (17)	0.044 (2)	0.0388 (18)	−0.0071 (15)	0.0023 (14)	−0.0011 (15)
Si1	0.0305 (4)	0.0289 (5)	0.0362 (5)	0.0054 (4)	−0.0002 (3)	0.0007 (4)
C1	0.0345 (16)	0.0283 (16)	0.0352 (17)	−0.0044 (13)	−0.0035 (13)	−0.0021 (13)
C2	0.0264 (15)	0.0319 (17)	0.0372 (17)	−0.0055 (13)	−0.0018 (12)	−0.0062 (13)
C3	0.0367 (18)	0.044 (2)	0.0389 (19)	−0.0070 (15)	0.0050 (14)	−0.0105 (15)
C4	0.047 (2)	0.056 (2)	0.0352 (19)	−0.0149 (18)	0.0078 (15)	−0.0107 (17)
C5	0.065 (2)	0.049 (2)	0.0327 (18)	−0.0198 (19)	0.0001 (16)	0.0003 (16)
C6	0.049 (2)	0.039 (2)	0.0379 (19)	−0.0090 (16)	−0.0025 (15)	0.0006 (15)
C7	0.0319 (17)	0.039 (2)	0.055 (2)	0.0053 (14)	−0.0033 (15)	0.0023 (16)
C8	0.047 (2)	0.0363 (19)	0.046 (2)	0.0047 (16)	0.0006 (16)	−0.0024 (15)
C11	0.0278 (15)	0.0296 (16)	0.0362 (17)	0.0029 (13)	−0.0001 (12)	−0.0014 (13)
C12	0.0266 (14)	0.0306 (16)	0.0317 (16)	0.0007 (13)	−0.0014 (12)	−0.0068 (13)
C13	0.0303 (17)	0.041 (2)	0.0438 (19)	0.0093 (14)	−0.0010 (14)	−0.0065 (15)
C14	0.0413 (19)	0.041 (2)	0.046 (2)	0.0183 (16)	−0.0054 (15)	−0.0008 (16)
C15	0.050 (2)	0.051 (2)	0.0377 (19)	0.0160 (17)	−0.0006 (15)	0.0059 (16)
C16	0.0404 (18)	0.048 (2)	0.0363 (18)	0.0161 (16)	0.0052 (14)	0.0047 (15)

### Geometric parameters (Å, °)

**Table:**

Si1A—C7A	1.865 (4)	Si1—C7	1.867 (3)
Si1A—C8A	1.868 (4)	Si1—C8	1.868 (4)
Si1A—C1A	1.882 (3)	Si1—C1	1.884 (3)
Si1A—C11A	1.887 (3)	Si1—C11	1.892 (3)
C1A—C6A	1.396 (5)	C1—C6	1.396 (5)
C1A—C2A	1.421 (5)	C1—C2	1.424 (4)
C2A—C3A	1.399 (5)	C2—C3	1.399 (5)
C2A—C12A	1.495 (5)	C2—C12	1.488 (4)
C3A—C4A	1.386 (5)	C3—C4	1.387 (5)
C3A—H3A	0.9500	C3—H3	0.9500
C4A—C5A	1.384 (6)	C4—C5	1.393 (6)
C4A—H4A	0.9500	C4—H4	0.9500
C5A—C6A	1.389 (5)	C5—C6	1.396 (5)
C5A—H5A	0.9500	C5—H5	0.9500
C6A—H6A	0.9500	C6—H6	0.9500
C7A—H7A1	0.9800	C7—H7A	0.9800
C7A—H7A2	0.9800	C7—H7B	0.9800
C7A—H7A3	0.9800	C7—H7C	0.9800
C8A—H8A1	0.9800	C8—H8A	0.9800
C8A—H8A2	0.9800	C8—H8B	0.9800
C8A—H8A3	0.9800	C8—H8C	0.9800
C11A—C16A	1.396 (5)	C11—C16	1.392 (5)
C11A—C12A	1.417 (4)	C11—C12	1.417 (4)
C12A—C13A	1.401 (5)	C12—C13	1.399 (4)
C13A—C14A	1.383 (5)	C13—C14	1.381 (5)
C13A—H13A	0.9500	C13—H13	0.9500
C14A—C15A	1.392 (5)	C14—C15	1.391 (5)
C14A—H14A	0.9500	C14—H14	0.9500
C15A—C16A	1.395 (5)	C15—C16	1.394 (5)
C15A—H15A	0.9500	C15—H15	0.9500
C16A—H16A	0.9500	C16—H16	0.9500
			
C7A—Si1A—C8A	108.95 (18)	C7—Si1—C8	108.96 (17)
C7A—Si1A—C1A	115.01 (17)	C7—Si1—C1	113.95 (16)
C8A—Si1A—C1A	112.61 (16)	C8—Si1—C1	114.13 (16)
C7A—Si1A—C11A	114.04 (15)	C7—Si1—C11	114.35 (15)
C8A—Si1A—C11A	114.28 (16)	C8—Si1—C11	113.64 (15)
C1A—Si1A—C11A	91.21 (14)	C1—Si1—C11	91.05 (14)
C6A—C1A—C2A	118.6 (3)	C6—C1—C2	118.7 (3)
C6A—C1A—Si1A	132.0 (3)	C6—C1—Si1	131.9 (3)
C2A—C1A—Si1A	109.4 (2)	C2—C1—Si1	109.4 (2)
C3A—C2A—C1A	119.9 (3)	C3—C2—C1	120.1 (3)
C3A—C2A—C12A	125.1 (3)	C3—C2—C12	124.9 (3)
C1A—C2A—C12A	115.0 (3)	C1—C2—C12	114.9 (3)
C4A—C3A—C2A	119.8 (4)	C4—C3—C2	119.8 (3)
C4A—C3A—H3A	120.1	C4—C3—H3	120.1
C2A—C3A—H3A	120.1	C2—C3—H3	120.1
C5A—C4A—C3A	120.7 (3)	C3—C4—C5	120.7 (3)
C5A—C4A—H4A	119.6	C3—C4—H4	119.7
C3A—C4A—H4A	119.6	C5—C4—H4	119.7
C4A—C5A—C6A	120.1 (4)	C4—C5—C6	119.9 (3)
C4A—C5A—H5A	120.0	C4—C5—H5	120.0
C6A—C5A—H5A	120.0	C6—C5—H5	120.0
C5A—C6A—C1A	120.8 (4)	C1—C6—C5	120.7 (4)
C5A—C6A—H6A	119.6	C1—C6—H6	119.7
C1A—C6A—H6A	119.6	C5—C6—H6	119.7
Si1A—C7A—H7A1	109.5	Si1—C7—H7A	109.5
Si1A—C7A—H7A2	109.5	Si1—C7—H7B	109.5
H7A1—C7A—H7A2	109.5	H7A—C7—H7B	109.5
Si1A—C7A—H7A3	109.5	Si1—C7—H7C	109.5
H7A1—C7A—H7A3	109.5	H7A—C7—H7C	109.5
H7A2—C7A—H7A3	109.5	H7B—C7—H7C	109.5
Si1A—C8A—H8A1	109.5	Si1—C8—H8A	109.5
Si1A—C8A—H8A2	109.5	Si1—C8—H8B	109.5
H8A1—C8A—H8A2	109.5	H8A—C8—H8B	109.5
Si1A—C8A—H8A3	109.5	Si1—C8—H8C	109.5
H8A1—C8A—H8A3	109.5	H8A—C8—H8C	109.5
H8A2—C8A—H8A3	109.5	H8B—C8—H8C	109.5
C16A—C11A—C12A	118.5 (3)	C16—C11—C12	118.4 (3)
C16A—C11A—Si1A	132.1 (2)	C16—C11—Si1	132.3 (2)
C12A—C11A—Si1A	109.4 (2)	C12—C11—Si1	109.3 (2)
C13A—C12A—C11A	120.2 (3)	C13—C12—C11	119.9 (3)
C13A—C12A—C2A	124.8 (3)	C13—C12—C2	124.8 (3)
C11A—C12A—C2A	114.9 (3)	C11—C12—C2	115.3 (3)
C14A—C13A—C12A	120.0 (3)	C14—C13—C12	120.3 (3)
C14A—C13A—H13A	120.0	C14—C13—H13	119.9
C12A—C13A—H13A	120.0	C12—C13—H13	119.9
C13A—C14A—C15A	120.4 (3)	C13—C14—C15	120.7 (3)
C13A—C14A—H14A	119.8	C13—C14—H14	119.7
C15A—C14A—H14A	119.8	C15—C14—H14	119.7
C14A—C15A—C16A	119.9 (3)	C14—C15—C16	119.2 (3)
C14A—C15A—H15A	120.0	C14—C15—H15	120.4
C16A—C15A—H15A	120.0	C16—C15—H15	120.4
C15A—C16A—C11A	120.9 (3)	C11—C16—C15	121.6 (3)
C15A—C16A—H16A	119.5	C11—C16—H16	119.2
C11A—C16A—H16A	119.5	C15—C16—H16	119.2
			
C7A—Si1A—C1A—C6A	−65.4 (4)	C7—Si1—C1—C6	−60.0 (4)
C8A—Si1A—C1A—C6A	60.2 (4)	C8—Si1—C1—C6	66.1 (3)
C11A—Si1A—C1A—C6A	177.2 (3)	C11—Si1—C1—C6	−177.3 (3)
C7A—Si1A—C1A—C2A	116.2 (2)	C7—Si1—C1—C2	119.8 (2)
C8A—Si1A—C1A—C2A	−118.1 (2)	C8—Si1—C1—C2	−114.2 (2)
C11A—Si1A—C1A—C2A	−1.1 (2)	C11—Si1—C1—C2	2.4 (2)
C6A—C1A—C2A—C3A	2.2 (5)	C6—C1—C2—C3	−2.6 (4)
Si1A—C1A—C2A—C3A	−179.2 (2)	Si1—C1—C2—C3	177.6 (2)
C6A—C1A—C2A—C12A	−176.6 (3)	C6—C1—C2—C12	176.4 (3)
Si1A—C1A—C2A—C12A	2.0 (3)	Si1—C1—C2—C12	−3.4 (3)
C1A—C2A—C3A—C4A	−0.5 (5)	C1—C2—C3—C4	0.7 (5)
C12A—C2A—C3A—C4A	178.1 (3)	C12—C2—C3—C4	−178.1 (3)
C2A—C3A—C4A—C5A	−0.7 (5)	C2—C3—C4—C5	0.9 (5)
C3A—C4A—C5A—C6A	0.3 (6)	C3—C4—C5—C6	−0.7 (5)
C4A—C5A—C6A—C1A	1.4 (5)	C2—C1—C6—C5	2.8 (5)
C2A—C1A—C6A—C5A	−2.6 (5)	Si1—C1—C6—C5	−177.5 (3)
Si1A—C1A—C6A—C5A	179.2 (3)	C4—C5—C6—C1	−1.2 (5)
C7A—Si1A—C11A—C16A	63.3 (4)	C7—Si1—C11—C16	59.2 (4)
C8A—Si1A—C11A—C16A	−62.9 (4)	C8—Si1—C11—C16	−66.7 (4)
C1A—Si1A—C11A—C16A	−178.5 (3)	C1—Si1—C11—C16	176.2 (3)
C7A—Si1A—C11A—C12A	−118.2 (2)	C7—Si1—C11—C12	−117.9 (2)
C8A—Si1A—C11A—C12A	115.5 (2)	C8—Si1—C11—C12	116.1 (2)
C1A—Si1A—C11A—C12A	0.0 (2)	C1—Si1—C11—C12	−0.9 (2)
C16A—C11A—C12A—C13A	0.9 (5)	C16—C11—C12—C13	−0.7 (5)
Si1A—C11A—C12A—C13A	−177.8 (2)	Si1—C11—C12—C13	176.9 (2)
C16A—C11A—C12A—C2A	179.9 (3)	C16—C11—C12—C2	−178.4 (3)
Si1A—C11A—C12A—C2A	1.2 (3)	Si1—C11—C12—C2	−0.8 (3)
C3A—C2A—C12A—C13A	−1.9 (5)	C3—C2—C12—C13	4.2 (5)
C1A—C2A—C12A—C13A	176.8 (3)	C1—C2—C12—C13	−174.7 (3)
C3A—C2A—C12A—C11A	179.1 (3)	C3—C2—C12—C11	−178.2 (3)
C1A—C2A—C12A—C11A	−2.2 (4)	C1—C2—C12—C11	2.8 (4)
C11A—C12A—C13A—C14A	0.5 (5)	C11—C12—C13—C14	0.1 (5)
C2A—C12A—C13A—C14A	−178.4 (3)	C2—C12—C13—C14	177.6 (3)
C12A—C13A—C14A—C15A	−1.3 (5)	C12—C13—C14—C15	0.1 (5)
C13A—C14A—C15A—C16A	0.7 (6)	C13—C14—C15—C16	0.3 (6)
C14A—C15A—C16A—C11A	0.7 (5)	C12—C11—C16—C15	1.1 (5)
C12A—C11A—C16A—C15A	−1.5 (5)	Si1—C11—C16—C15	−175.9 (3)
Si1A—C11A—C16A—C15A	176.8 (3)	C14—C15—C16—C11	−0.9 (6)
